# An Ultra-Fast Validated Green UPLC-MS/MS Approach for Assessing Revumenib in Human Liver Microsomes: In Vitro Absorption, Distribution, Metabolism, and Excretion and Metabolic Stability Evaluation

**DOI:** 10.3390/medicina60121914

**Published:** 2024-11-21

**Authors:** Mohamed W. Attwa, Ali S. Abdelhameed, Adnan A. Kadi

**Affiliations:** Department of Pharmaceutical Chemistry, College of Pharmacy, King Saud University, Riyadh 11451, Saudi Arabia; asaber@ksu.edu.sa (A.S.A.); akadi@ksu.edu.sa (A.A.K.)

**Keywords:** revumenib, greenness, in vitro half-life, UPLC-MS/MS, metabolic stability

## Abstract

*Background and Objectives:* Revumenib (SNDX-5613) is a powerful and specific inhibitor of the menin–KMT2A binding interaction. It is a small molecule that is currently being researched to treat KMT2A-rearranged (KMT2Ar) acute leukemias. Revumenib (RVB) has received Orphan Drug Designation from the US FDA for treating patients with AML. It has also been granted Fast Track designation by the FDA for treating pediatric and adult patients with R/R acute leukemias that have a KMT2Ar or NPM1 mutation. *Materials and Methods*: The target of this research was to create a fast, precise, green, and extremely sensitive UPLC-MS/MS technique for the estimation of the RVB level in human liver microsomes (HLMs), employing an ESI source. The validation procedures were carried out in accordance with the bioanalytical technique validation requirements established by the US Food and Drug Administration that involve linearity, selectivity, precision, accuracy, stability, matrix effect, and extraction recovery. The outcome data of the validation features of the UPLC-MS/MS approach were acceptable according to FDA guidelines. RVB parent ions were formed in the positive ESI source and its two fragment ions were estimated employing multiple reaction monitoring (MRM) mode. The separation of RVB and encorafenib was achieved using a C8 column (2.1 mm, 50 mm, and 3.5 µm) and isocratic mobile phase. *Results:* The RVB calibration curve linearity ranged from 1 to 3000 ng/mL (y = 0.6515x − 0.5459 and *R*^2^ = 0.9945). The inter-day precision and accuracy spanned from −0.23% to 11.33%, while the intra-day precision and accuracy spanned from −0.88% to 11.67%, verifying the reproducibility of the UPLC-MS/MS analytical technique. The sensitivity of the developed methodology demonstrated its capability to quantify RVB levels at an LOQ of 0.96 ng/mL. The AGREE score was 0.77, confirming the greenness of the current method. The low in vitro t_1/2_ (14.93 min) and high intrinsic clearance (54.31 mL/min/kg) of RVB revealed that RVB shares similarities with medications that have a high extraction ratio. *Conclusions*: The present LC-MS/MS approach is considered the first analytical approach with the application of metabolic stability assessment for RVB estimation in HLMs. These methods are essential for advancing the development of new pharmaceuticals, particularly in enhancing metabolic stability.

## 1. Introduction

Cancer is widely documented as the primary worldwide cause of death, defined as the uncontrolled proliferation of cells in a certain organ of the human body. Furthermore, it possesses the ability to metastasize to various organs, as a result of genetic idiosyncrasies that impede the regulation of several biological mechanisms, therefore enabling the proliferation of malignant cells [1]. Among various forms of cancer, leukemia is one of the most severe and critical health challenges confronting the globe today [2]. Globally, almost 2.43 million individuals are affected by leukemia [3]. It ranks as the 15th most frequently diagnosed malignancy and the 11th leading cause of cancer, representing 3.2% of all new cancer cases and 3.9% of all cancer fatalities [4]. The prevalence of leukemia is greater among older individuals, resulting in a higher burden in affluent nations compared with developing countries [5]. Leukemia terminology denotes the malignancy of the bone marrow, where the genesis of blood cells occurs. Leukemia is further categorized as myeloid leukemia, which affects various blood cells excluding immune cells, or lymphoid leukemia, which involves immune cells such as B cells, T cells, and NK cells. Based on its complications, the cancer is classified into acute or chronic leukemia. Of these two cases, acute leukemia disseminates rapidly, necessitating prompt treatment [6]. Acute myeloid leukemia (AML) is a prevalent and rapidly progressing hematological malignancy originating from myeloblasts. In individuals afflicted with AML, white blood cells multiply abnormally, rendering them ineffective in combating both existing and future infections [7,8].

**Figure 1:**
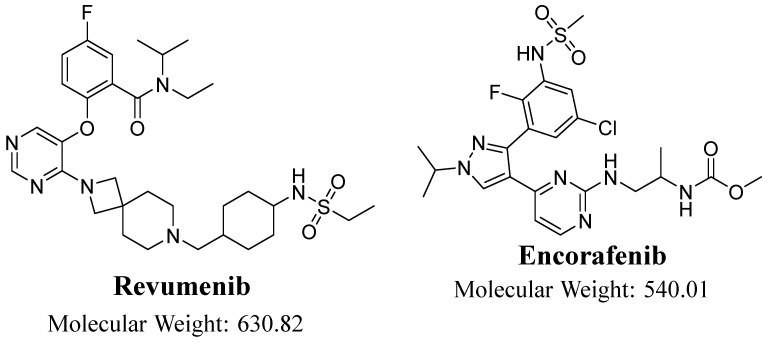
Chemical structure of the target analyte, revumenib, and the encorafenib that was used as an internal standard in the UPLC-MS/MS analysis of RVB.

Molecular targeted therapy employing therapeutic monoclonal antibodies or small molecule medicines that function as signal transduction inhibitors has been a crucial foundation of precision medicine for cancer treatment [9]. These methodologies are currently employed clinically as the primary treatments for several forms of human malignancies [10]. In contrast to conventional chemotherapy, targeted therapeutic medicines exhibit effective anticancer properties with reduced adverse effects [11]. The acquired or intrinsic drug resistance directly impacts the prognosis and the survival of cancer patients [12]. The evolution of drug resistance poses a significant challenge to molecular targeted therapy, prompting the exploration of various ways to enhance therapeutic efficacy by mitigating this resistance [13]. Menin functions as a scaffold protein, and its interaction with KMT2A results in the activation of genes linked to the development of leukemia, such as HOX and MEIS1. Revumenib (RVB), also known as SNDX-5613, treats KMT2A-rearranged (KMT2Ar) acute leukemias by binding to menin’s binding pocket and displacing KMT2A. This action effectively shuts down the HOX and MEIS genes, leading to the cessation of leukemic cell proliferation. RVB was created by Syndax Pharmaceuticals, Inc. (Waltham, MA, USA) (Figure 1). RVB is classified as a pioneering menin inhibitor and is currently being developed to treat relapsed or refractory (R/R) KMT2A-rearranged acute leukemias, which include both acute myeloid leukemia (AML) and acute lymphoblastic leukemia (ALL), as well as NPM1-mutant AML.

RVB has received Orphan Drug Designation from the US FDA for treating patients with AML. It has also been granted Fast Track status by the FDA for treating pediatric and adult patients with R/R acute leukemias that have a NPM1 mutation or KMT2A rearrangement. RVB received Breakthrough Therapy Designation from the FDA for treating pediatric and adult patients with R/R acute leukemia that had a KMT2Ar [14]. The FDA has awarded priority review status to the new drug application (NDA) for RVB. The NDA submission is currently undergoing evaluation within the FDA’s Real-Time Oncology Review Program (RTOR) and has been given a target action date of 26 September 2024, as per the Prescription Drug User Fee Act (PDUFA) [15].

The primary objective of this investigation was to examine the metabolic stability of RVB in a controlled laboratory setting, employing a UPLC-MS/MS technique [16,17,18]. The creation of a rapid, environmentally safe, and extremely sensitive analytical UPLC-MS/MS methodology for determining RVB in various matrices including human liver microsomes (HLMs) has immense importance. Precise assessment of a specific medicine (RVB in this investigation) is crucial for the surveillance of its therapeutic impacts (TDM). Furthermore, it is crucial to comprehend the relationship between the activity of RVB and its level in order to ensure the reliable and secure use of this medication by patients. A thorough examination of the existing literature indicates a notable scarcity of published studies on the quantification of RVB in different compounds. Until now, there has been a lack of documented assessment of the metabolic instability of RVB in HLMs via UPLC-MS/MS methodology. Evaluating the metabolic stability of RVB in HLMs is essential for comprehending the kinetics of its metabolic pathways and removal [19,20].

The metabolic stability of a drug is a measure of its susceptibility to experience metabolic routes. This is determined by its in vitro half-life (t_1/2_) and intrinsic clearance (Cl_int_). The term “t_1/2_” denotes the duration required for 50% of the original pharmacologically active drug to undergo biotransformation. Cl_int_ represents the hepatic capability to metabolize a medical drug that is currently in the circulatory system. The target of this investigation was to establish a fast, sensitive, specific, and eco-friendly UPLC-MS/MS technique for assessing the metabolic stability of RVB in HLMs [21].

Lately, there has been rising interest in the field of green analytical chemistry (GAC), which aims to tackle the existence of dangerous chemicals, minimize waste production, and lower energy usage with the support of various analytical techniques [22,23]. Various measurement approaches have been utilized to estimate levels of environmental sustainability based on various analytical findings, with the goal of attaining these targets. These have included various approaches such as Analytical Green-ness Metric Approach (AGREE), Green Analytical Procedures Index (GAPI), Analytical Eco-Scale (AES), and the National Environmental Methods Index (NEMI) [22]. The GAPI, NEMI, and AES approaches demonstrat a dependence on certain GAC standards, as depicted. This study utilized the “AGREE” methodology to evaluate the extent of ecological sustainability by analyzing 12 GAC criteria and assigning numerical data to each [24].

The UPLC-MS/MS technique used a mobile phase of consistent composition and had a total runtime of 1 min, leading to a highly efficient analytical procedure. By introducing a flow rate at 0.6 mL/min and reducing the amount of ACN to 45%, the previous system’s environmentally friendly attributes were greatly improved. In addition, the method used in this study showed a clear and consistent relationship across a wide range of values, ranging from 1 to 3000 ng/mL. The present method was implemented with the objective of decreasing expenses and limiting the duration. The in vitro t_1/2_ and Cl_int_ of RVB were evaluated in this research using the present UPLC-MS/MS system, following the techniques described in earlier research papers [25]. These methodologies can be employed to calculate metabolic rate in living beings, employing three distinct models: parallel tube, venous equilibrium, and dispersion. The Cl_int_ and t_1/2_ of RVB were estimated using an in vitro methodology, namely, the well-stirred model [26,27]. The application of this model is commonly noticed in studies on drug metabolism due to its effortlessness and easy implementation.

## 2. Methodology

### 2.1. Materials

The study utilized revumenib (RVB) and encorafenib (ENF) as solid medicines, which were obtained at analytical grade (AR). The ongoing work focuses on the analysis of two specific compounds, RVB (also referred to as SNDX-5613) and ENF (also known as LGX818). The analytes were sourced from MedChem Corporation, a reputable pharmaceutical company headquartered in Princeton, NJ, USA. The purity of RVB was determined to be 99.88%, whereas ENF exhibited a purity of 99.63%. The chemicals utilized in this investigation, ammonium formate (NH_4_COOH), formic acid (HCOOH), acetonitrile (ACN), methanol (CH_3_OH), and HLMs (20 mg/mL), were purchased from Sigma Aldrich company, situated in St. Louis, MI, USA. The HLMs were shipped on dry ice to ensure the preservation of their integrity and then stored in a deep freezer at −78 °C until the time of use. The solvents (ACN, H_2_O, HCOOH, and CH_3_OH) employed in the UPLC-MS/MS approach in this investigation were of HPLC grade, indicating their outstanding performance in liquid chromatography.

### 2.2. Instruments

A Milli-Q apparatus, established by Millipore Corporation in Billerica, MA, USA, was utilized for the water filtration process to produce HPLC-grade water. This investigation utilized a UPLC-MS/MS system (Waters company, Milford, MA, USA) composed of two main parts including an Acquity UPLC (H10UPH) and an Acquity TQD MS (QBB1203). The vacuum in the TQD mass detector was established employing a vacuum pump manufactured by Sogevac Company, located in Murrysville, PA, USA. The UPLC-MS/MS system utilized MassLynx software (Version 4.1, SCN 805). The gadget was utilized to conduct an inclusive study and characterize the analytical peaks in the RVB and ENF targets. The targets were obtained from the HLM incubation matrix using protein precipitation. QuanLynx, an application manager included with Waters MassLynx Software (Version 4.1, SCN 805), was employed for quantitative analysis in the current study. The MassLynx software package has two important software constituents, namely, QuanLynx and IntelliStart^®^ (Milford, MA, USA). QuanLynx (Version 4.1, SCN 805) was used for rapid processing of the acquired mass spectrometry data of sample, due to the speed of acquisition. The optimization of mass spectrometry (MS) parameters for the analytes RVB and ENF was performed with the IntelliStart^®^ program (Version 4.1, SCN 805), part of the MassLynx 4.1 software suite (version 4.1, SCN 805). The application of nitrogen gas improved the droplets’ evaporation in the ionization source (ESI) of the mobile phase. The gas was produced via a nitrogen generator from Peak Scientific Corporation, located in Scotland, UK. The RVB and ENF ions decomposed within the collision cell (second quadrupole), producing their resultant ions. The fragmentation technique was improved using argon gas with a purity of 99.999% as the collision gas.

### 2.3. DVB In Silico ADME Profile

SwissADME in silico software (version 1), developed by the Swiss Institute of Bioinformatics (Switzerland), was utilized to propose the ADME features of RVB. The software can be accessed online through the web link at http://www.swissadme.ch/ (accessed on 1 August 2024).

### 2.4. UPLC-MS/MS Analytical Features

Prior to conducting the LC-MS/MS experiment, the MS analyzer was tuned with a tuning solution to confirm its accuracy. The MRM tune files for analytes (RVB and ENF) were freshly prepared to adjust any error in *m*/*z* detection. The UPLC-MS/MS specific features were fine-tuned to maximize the resolution and sensitivity of the RVB and ENF chromatographic peaks. A comprehensive investigation was carried out to enhance the analytical properties of the HPLC approach by considering several parameters such as the pH level, mobile phase, and characteristics of the stationary phase. The aim of these tuning steps was to improve the separation and sensitivity of the analytes’ peaks associated with the targets, RVB and ENF. The mobile phase included two lines (binary system): line A, comprising an aqueous solution containing 0.1% formic acid in H_2_O with a pH of 3.2, constituting 55%; and line B, consisting of ACN, constituting 45%. The recorded flow rate was determined to be 0.6 mL/min. At a pH of approximately 3.2, the utilization of a 10 mM NH_4_COOH solution resulted in the occurrence of tailing in the RVB chromatographic peak, along with an elongated run time. Once the ratio of ACN exceeded 45%, the chromatograms of RVB and ENF had significant peaks that overlapped with each other. On the other hand, a smaller percentage of ACN resulted in a lengthier elution time. The column used was an Eclipse Plus C8 column (2.1 mm, 50 mm, and 3.5 µm). The column was maintained at a temperature of 22.0 ± 2.0 °C, and a volume of 5.0 μL was injected.

The ESI source (the interface) was utilized in the positive charge mode to promote generation of positive ions, as the RVB and ENF targets enclosed nitrogen atoms capable of trapping protons, leading to ion production. The RF lens, extractor, and capillary voltages were set to 0.1 V, 3.0 V, and 4 KV, respectively. The ESI source utilized nitrogen gas at 350 °C and a flow rate of 100 L/hr for the purpose of drying. The dissociation of the RVB and ENF in the collision cell was achieved by using argon gas at 0.14 mL/min as the dissociation gas. The IntelliStart^®^ application was successfully used to optimize the MS settings for RVB (C_32_H_47_FN_6_O_4_S) and ENF (C_22_H_27_ClFN_7_O_4_S). This was achieved by directly incorporating RVB and ENF (10 µg/mL) into the mobile phase.

Multiple reaction monitoring (MRM) resembles selected reaction monitoring (SRM) but facilitates the concurrent observation of numerous parent–product ion transitions in a single investigation [28]. This multiplexing feature improves throughput and efficiency in quantification experiments. In SRM/MRM, this procedure is augmented by tandem mass spectrometry (MS/MS), wherein parent ions of interest (the analytes under investigation) are isolated in the first quadrupole (Q1) and dissociated in a collision cell. Then, specific fragment ions are identified and analyzed in the third quadrupole (Q3). This precursor-to-product ion transition is meticulously regulated, enabling extremely selective and sensitive detection of target analytes within intricate sample matrices. The MRM approach was utilized as the mass analysis method to calculate the RVB and ENF. The adoption of this methodology led to an enhanced degree of exactness and correctness for the UPLC-MS/MS approach that was established. The duration for the reaction monitoring from parent ions to fragment ions in RVB and ENF was measured to be 0.025 s. The two MRM transitions for ENF (0.55 to 1.0 min) were from 540 to 359 and from 540 to 116. The cone voltage (CV) was set at 36 V and 32 V for ENF, with collision energy (CE) of 56 eV. For RVB (0.0 to 0.55 min), the mass transitions were from 631 to 110 and from 631 to 95. For RVB, the CV was set at 82 V, with CE of 56 eV.

### 2.5. RVB and ENF Working Dilutions

The greatest solubility of RVB and ENF was seen in DMSO at ≥25 mg/mL (39.63 mM; using freshly opened DMSO, as the presence of hygroscopic DMSO significantly affects the solubility of RVB) and 50 mg/mL (92.59 mM; reached only after ultrasonic processing), respectively. Hence, the RVB and ENF stock solutions at 1 mg/mL level were solubilized in DMSO. The adjusted mobile phase was used to create RVB working solutions at 100, 10, and 1 µg/mL and ENF at 10 µg/mL, using a progressive dilution technique. The RVB and ENF stock preparations were initially made at 1 mg/mL, and subsequent dilutions were performed.

### 2.6. Establishing of RVB Calibration Standards

Preceding to initiating the validation step for the current UPLC-MS/MS process, the HLMs matrix was deactivated by the addition of 2% DMSO solution and incubated at 50 °C for 5 min. The objective of adopting this prudent method was to mitigate the potential metabolic influences of HLMs [29,30,31] on the RVB and ENF. A special validation matrix was prepared for HLMs to assess the metabolic stability of RVB. This procedure entailed the incorporation of 30 µL of inactive HLMs, at a concentration of 1 mg/mL protein, into 1 mL of the HLM metabolic matrix. A metabolic buffer solution was prepared using 0.1 M sodium phosphate buffer solution (pH of 7.4) that contained 1 mM of NADPH and 3.3 mM of MgCl_2_. The target of employing this procedure was to replicate the conditions of practical in vitro incubation for experimental purposes. The RVB (WK2 and WK3) experienced a multistage dilution procedure utilizing the inactive HLM matrix to produce RVB CSs. A total of nine CSs were prepared (ranging from 1 to 3000 ng/mL). Furthermore, four QCs were implemented: 3 ng/mL (lower QC, LQC), 1 ng/mL (lower limit of quantification, LLOQ), 900 ng/mL (middle QC, MQC), and 2400 ng/mL (high QC, HQC). During the dilution procedure, stringent procedures were utilized to maintain the HLM matrix concentration over 90%. The objective of this activity was to mitigate the potential effects of matrix dilution, thus simulating the circumstances of in vitro incubation. The QCs served as samples with unknown concentration, and their data were ascertained using the linear regression equation derived from the parallel injected RVB CSs. One hundred µL ENF WK solution (10,000 ng/mL) was administered to all RVB CSs and QCs.

### 2.7. The Recovery of RVB and ENF from the HLMs

The extraction technique of protein precipitation was used to extract the RVB and ENF from the HLMs. The ACN solvent effectively served its purpose in this procedure by inhibiting and isolating proteins inside the HLM matrix. Subsequently, 2 mL of ACN was introduced into each of the RVB QCs and CSs, and the mixture experienced nonstop stirring for 5 min in order to enhance the extraction efficiency of RVB and ENF. Afterwards, the combination was centrifuged at a speed of 14,000 rpm and 4 °C for 12 min. The centrifugation methodology was employed to precipitate proteins and obtain a clarified condition in the upper clear supernatant. Filtration was employed with all incubates to achieve reliability and sample suitability for injection into the UPLC-MS/MS instrument. The procedure employed a 0.22 µm syringe filter. The refined extracts were added to vials in anticipation of being loaded into a UPLC-MS/MS instrument. In order to conduct the experiment, two control samples were generated. The first negative-control sample, referred to as the first control sample, consisted of HLMs. The second positive-control sample consisted of HLMs that were enhanced with ENF (IS). The above-mentioned methodologies were replicated to confirm the lack of any influence exerted by the contents of HLMs when differentiating between RVB and ENF. The procedure of constructing a calibration curve for an RVB entails plotting the given RVB data on the *x*-axis, while the *y*-axis represents the peak area ratio of RVB to ENF. The linearity span range of the generated RVB CSs was evaluated by examining the validation parameters and the regression equation (y = ax + b; *R*^2^) of the constructed linear calibration curve.

### 2.8. Validation Features of the Established UPLC-MS/MS Approach

The validation features of the UPLC-MS/MS approach [24] were conducted following ICH Q2(R1) recommendations [32]. The validation methodology consisted of evaluating multiple characteristics such as precision, linearity, stability, accuracy, specificity, extraction recovery, sensitivity, and matrix effect [33,34,35,36].

#### 2.8.1. Specificity

The specificity validation parameter of the UPLC-MS/MS method was assessed by injecting 6 sets of blank HLMs matrix samples after the protein precipitation, which was the selected extraction technique. The filtered extracts were loaded into a UPLC-MS/MS system and analyzed to detect any possible intervention from constituent peaks occurring from the HLM matrix that could affect the analytical peaks’ (RVB or ENF) retention time. Afterwards, a comparative analysis was carried out to evaluate the gathered data relating to the spiked HLM samples containing RVB and ENF. The MRM analyzer mode was utilized to alleviate the lingering impacts of the RVB and ENF in the mass analyzer detector. The validation feature was attained by examining results obtained from the negative control samples of HLMs, revealing deficiencies in RVB and ENF.

#### 2.8.2. Linearity and Sensitivity

Twelve calibration curves were created to assess the linearity range and sensitivity limit of the UPLC-MS/MS system. The construction of these curves involved using seven CSs for RVB, which were executed in the HLM matrix, all completed in the same day. Afterwards, the linear regression equation of the linear calibration curve was employed to estimate the RVB in samples of unknowns. The limit of detection (LOD) and limit of quantification (LOQ) were assessed according to the guidelines as stated in the Pharmacopoeia. The study’s directions included determining the LOQ and the LOD by using the calibration curve slope and the intercept standard deviation (SD), as specified by Equations (1) and (2), respectively.

(1) LOQ=10∗SD of the interceptSlope

(2) LOD=3∗SD of the intercept Slope

The evaluation of linearity in the current UPLC-MS/MS approach included the use of statistical measurements, notably the least squares approach (y = ax + b) and the *R*^2^ value.

#### 2.8.3. Accuracy and Precision

The determination of precision and accuracy in the established UPLC-MS/MS approach necessitated performing numerous experiments in the same day for intra-day investigation, and over the following 3 days for inter-day investigation. A total of 6 groups of RVB QCs were utilized for inter-day analysis. However, the intra-day analysis employed 12 sets of RVB QCs. The precision and accuracy of the UPLC-MS/MS system were evaluated by calculating the percent error (%E) and percent relative SD (%RSD), respectively. The values were calculated using Equations (3) and (4):

(3) %E=(Calculated conc.—Supposed conc.)Supposedconc.∗100

(4) %RSD=SDMean

#### 2.8.4. Matrix Effect and Extraction Recovery

Matrix effects frequently arise from changes in the ionization efficiency of target analytes due to the existence of co-eluting substances within the same matrix. Matrix effects may manifest as a decrease in reaction (ion suppression) or as an augmentation in response (ion enhancement). Ion enhancement and suppression significantly impact the analytical performance of a technique [37]. Consequently, matrix effects must be assessed during the validation of an LC–MS technique. Since the initial observation of matrix effects, considerable efforts have been dedicated to elucidating the mechanisms and mitigating their impact [38]. To assess the impression of HLMs on the ionization degree of the targets (RVB or ENF), we created two distinct groups of LQC samples (six replicates). The HLM matrix was used as a matrix to prepare the group 1 (LQC samples) in this investigation. The HLM samples were supplemented using the RVB LQC at 3 ng/mL. Furthermore, an IS (ENF) was incorporated into the solution at 1000 ng/mL. Meanwhile, mobile phase was used as a matrix to prepare group 2 (LQC samples) in lieu of the metabolic matrix (HLMs). All samples from the two groups were extracted, purified and injected into the UPLC-MS/MS system; their data were collected and their levels were estimated. The normalized matrix effect (NME) for the IS was computed by Equation (5), and the ME values for RVB and ENF were determined by Equation (6):

(5) IS NME=ME of RVBME of ENF (IS)

(6) ME of RVB or ENF=Average peak area ratioSet1Set2×100

The determination of the retrieval of RVB from the HLMs and the examination of how HLMs impacted the extent of RVB parent ionization were made by applying 4 QCs. The validation of protein precipitation as the optimal extraction approach for RVB and ENF was performed by preparing 6 sets of 4 QCs in HLM matrix (B), and then relating them with 4 QCs generated using the selected mobile phase (A). The determination of extraction recovery percentages for RVB and ENF entailed the computation of the % value by multiplying the amount of B divided by A by 100.

#### 2.8.5. Stability

The objective of the present experiment was to assess the stability of RVB in HLM matrix and stock preparations in diverse research laboratory settings, including pre-analysis processes such as short- and long-term storage, three freeze–thaw cycles, and storage in an auto sampler. The QCs (LQC and HQC) were stored under autosampler conditions for about 24 h at 15 °C prior to being injected into the UPLC system for the purpose of assessing their stability in the autosampler. In order to assess the short-term stability, QC samples (including spiked HLMs) were stored at room temperature for a duration of 4 h before being loaded into the UPLC-MS/MS system. Three freeze–thaw cycles were conducted to assess the stability of the sample during the freezing (at −80 °C) and thawing (at normal room temperature) process. To evaluate the stability during prolonged storage circumstances, the samples were kept at −80 °C for 28 days before being analyzed in the UPLC-MS/MS instrument.

### 2.9. Evaluation of the In Vitro Metabolic Stability of RVB

The assessment of in vitro t_1/2_ and Cl_int_ of RVB entailed quantifying the residual level of RVB following its introduction to an in vitro incubation experiment involving the application of HLM matrix fortified with NADPH as a coenzyme and MgCl_2_. A four-step methodology was employed to conduct the in vitro incubation experiment. At the beginning, 1 µL of RVB (1 mM/mL) was mixed with HLM matrix to generate a concentration of 1 µM/mL and the mixture was allowed to pre-incubate [39]. The aforementioned method was carried out using a water bath that was kept at 37 °C using a thermostat for 10 min. At the start of the test, every sample was provided with a solution comprising 1 mM of NADPH. Afterwards, all samples were moved to a water bath that had a thermostat and the ability to shake at 37 °C. In the third phase of the current experiment, 100 µL of ENF (1000 ng/mL) was added before the ACN to act as a stopping agent. The target of the current approach was to achieve a consistent level of IS and reduce any probable effect of metabolic enzymatic pathways on the IS level. In the last step, referred to as the finish step, 2 mL volume of ACN was added at predetermined time breaks (0, 2.5, 7.5, 15, 20, 30, 40, 50, 60, and 70 min) to pause the metabolic activity and precipitate any surplus proteins. The first step of the extraction procedure for the RVB and ENF represented the primary stage, as designated in Section 2.6. A negative control sample was included to examine the effect of removing NADPH on the incubation of RVB with HLMs, using the tactic formerly described. The aim of the conducted test was to evaluate the potential impression of incubation circumstances and HLM matrix influences on the RVB level in the current in vitro metabolic stability study tests.

The remaining RVB level was determined by the regression equation attained from the concurrent CSs samples of RVB. The metabolic stability curve for RVB was generated by plotting the time breaks (*x*-axis) ranging from 0–70 min against the % of RVB level that persisted relative to the initial RVB level at time zero (100%) (*y*-axis). Hence, the interval of the metabolic curve (0–30 min) was used to generate the LN curve. The LN of RVB level was plotted with respect to the appropriate metabolic time points throughout the time span of 0–30 min. The rate constant (slope) for RVB was calculated by examining the gradient of the curve shown above. Subsequently, the slope was utilized to determine the t_1/2_ (in vitro t_1/2_ = ln2/ slope). The Cl_int_ value in mL/min/kg was established by referring to previous research [40]. Equation (7) necessitated the utilization of liver tissue mass (26 g) per kilogram of body weight and the matrix mass of HLMs (45 mg) per gram of liver tissue for the computation at hand [41]:

(7) $${Cl}_{int,}=0693\times \frac{1}{t_{½}(min.)}\times \frac{mL\;incubation}{mg\;protein}\times \frac{mg\;microsomal\;proteins}{g\;of\;liver\;weight}\times \frac{g\;liver}{Kg\;b.w.}$$

## 3. Results and Discussions

### 3.1. In Silico ADME Profile

**Figure 2:**
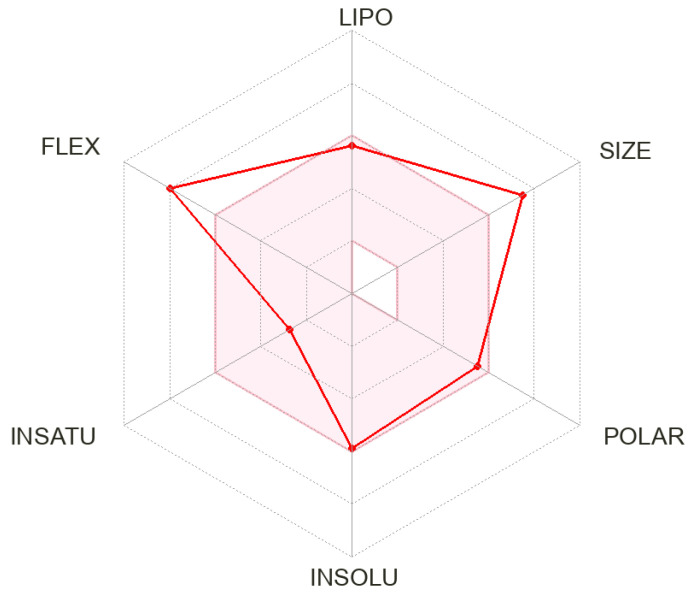
The RVB ADME radar chart produced from the in silico SwissADME program.

The ADME properties of RVB were determined by submitting the Smiles format of RVB (CCN(C(=O)c1cc(F)ccc1Oc1cncnc1N1CC2(C1)CCN(CC2)CC1CCC(CC1)NS(=O)(=O)CC)C(C)C) to the Swiss ADME website. A comprehensive evaluation was carried out to investigate the propensity of RVB to exhibit drug-like properties, through an analysis of its ADME (absorption, distribution, metabolism, and excretion) qualities. Following the log p value produced by the SwissADME program (version 1), it was found that RVB has a modest level of solubility in water (Log S = −5.89). Additionally, it is worth noting that the predictable pharmacokinetic parameter associated with the absorption procedure in the GIT indicated a reduced level of absorption, while there was no reported permeability across the BBB. The specified bioavailability score was 0.55. RVB is proposed to work as an inhibitor for certain cytochrome P450 enzymes, specifically CYP3A4, as well as P-glycoprotein, which serves as a substrate. The assertion posits that RVB does not exhibit inhibitory influences on other cytochrome P450 enzymes, including CYP2C9, CYP2D6, CYP1A2, and CYP2C19. The Log Kp value, which represents the skin’s permeability, was determined to be −7.07 cm/s. With respect to drug likeness, it complies with the Egan and Lipinski recommendations, but it contravenes the Veber guideline due to a violation in the number of rotors being greater than 10. Additionally, it violates the Muegge guideline as the molecular weight exceeds 600. Furthermore, it violates the Ghose guideline in three aspects: the molecular weight exceeds 480, the molecular refractivity exceeds 130, and the number of atoms exceeds 70. Figure 2 displays the ADME radar chart for RVB, together with the accompanying data presented in Table 1.

### 3.2. UPLC-MS/MS Method

Two different natures of stationary phase, using reversed phase (C8 and C18) columns and a normal phase (HILIC) column, were tested for the capability of separating the target analytes (RVB and ENF). Neither the RVB nor the ENF were separated or retained when using the HILIC column. Using a C8 column showed beneficial results as the type of reversed stationary phase. Nevertheless, the usage of the UPLC-MS/MS technique for the resolution of RVB and ENF resulted in the retention of the targets within the analytical system due to the implementation of a C18 column. The observed targets, namely RVB and ENF, displayed insufficient differentiation of the primary peak, elongated peak tails, and an extended elution time. Utilizing an Agilent Eclipse Plus C8 column (Agilent Technologies, Santa Clara, CA, USA) with specified attributes including an inner diameter of 2.1 mm, a length of 50 mm, and a particle size of 3.5 μm yielded positive outcomes with respect to retention duration and the morphology of chromatographic peaks. The UPLC-MS/MS approach included resolution of the desired analytes, RVB and ENF, employing a mobile phase with a steady content. The separation procedure was carried out at 0.6 mL/min for one minute. The RVB calibration curve obtained using the specified approach showed linearity over a span range of 1 to 3000 ng/mL. Table 2 reports a comprehensive collection of multiple trials carried out to enhance and ascertain the optimal attributes for extracting, segregating, and assessing RVB and ENF peaks. The crucial target of this research was to attain appropriate characteristics, for example, a distinct and effectively defined analytical peak shape, in addition to a decreased retention duration.

**Figure 3:**
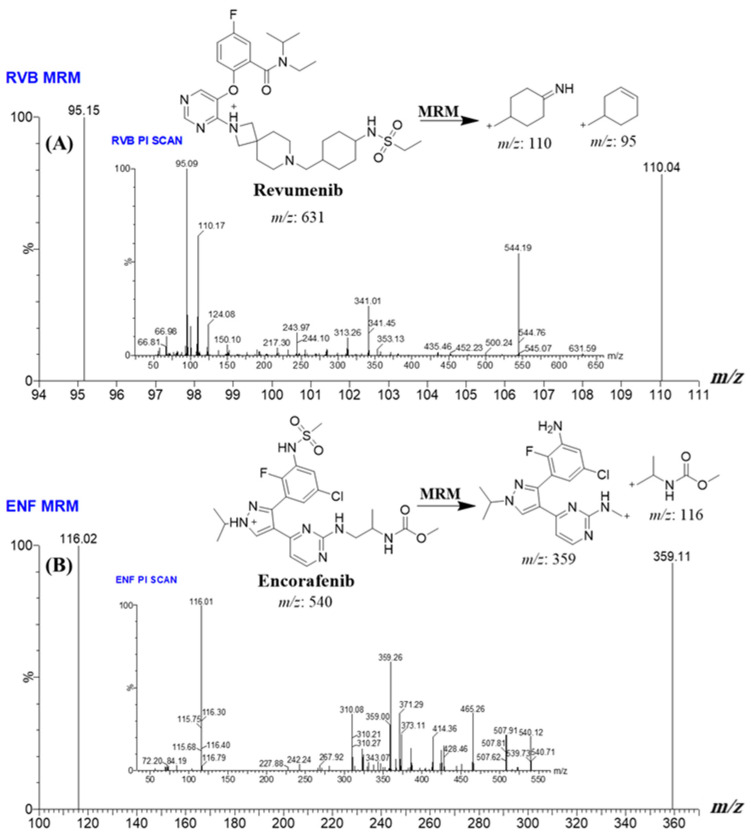
MRM spectrum showing PI mass scan of RVB as protonated molecular ion [M + H]^+^ (**A**) and MRM spectrum showing PI mass spectrum scan of ENF [M + H]^+^ (**B**). The probable dissociations behaviours are elucidated.

To increase the sensitivity and selectivity of the UPLC-MS/MS apparatus, the MRM analyzer mode was employed to precisely quantify and detect the concentrations of RVB and ENF. The goal of this investigation was to examine any plausible intervention induced by the matrix materials in the HLM matrix (Figure 3).

**Figure 4:**
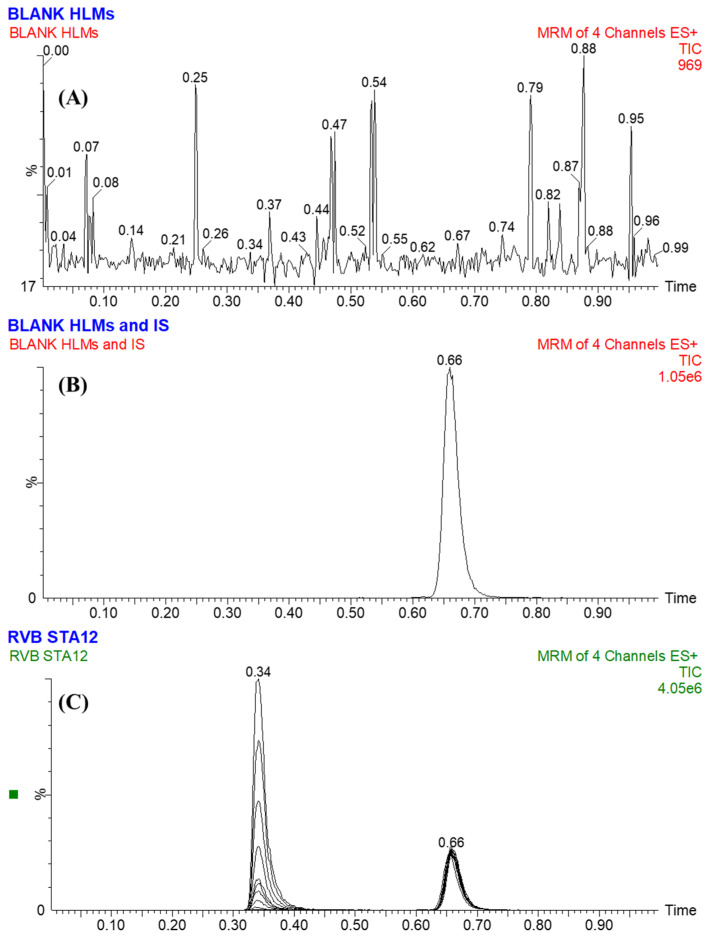
The MRM chromatogram of the first control sample (negative control HLMs) demonstrated the lack of any interference in the retention times of RVB and ENF (**A**). The MRM chromatogram of the second control sample, positive control (Blank HLMs combined with ENF at 1000 ng/mL) (**B**). The superimposed MRM chromatograms of the 9 RVB CSs, as well as the 3 QCs (**C**). The MRM chromatograms revealed analytical peaks conforming to RVB (at 0.34 min) and ENF at 1000 ng/mL and a retention time of 0.66 min).

The UPLC-MS/MS technique employed ENF as an internal standard (IS) to quantify RVB. This technique was dependent on three essential elements. The protein precipitation extraction procedure was demonstrated to be highly efficient in extracting both RVB and ENF analytes, resulting in a yield of 103.53 ± 4.46% with a RSD below 4.31% for RVB, and a yield of 101.61 ± 3.23% with an RSD below 3.18% for ENF. In addition, the RVB peak at 0.36 min and the ENF peak at 0.66 min were effectively obtained within a one-minute interval. These results demonstrate the usefulness of the present UPLC-MS/MS methodology as a capable and fast analytical tool. The utilized methodology not only effectively reduces the total running time of the process but also enhances the consumption of ACN, hereafter supporting the values of green chemistry. Additionally, it is important to know that individuals in certain medical environments do not consume both RVB and ENF simultaneously. So, the present UPLC-MS/MS approach is appropriate for performing pharmacokinetic and therapeutic drug monitoring (TDM) studies on RVB. No carry-over impact was detected in the generated MRM chromatograms of the first negative control (Figure 4A) and second positive control (Figure 4B) for RVB in the HLMs. Figure 4C shows the MRM chromatograms acquired from the MRM analysis of RVB CSs and ENF. The concentration range for RVB CSs spanned from 1 to 3000 ng/mL, while for ENF it was 1000 ng/mL.

### 3.3. Validation Features of the Established UPLC-MS/MS System

#### 3.3.1. Specificity

**Figure 5:**
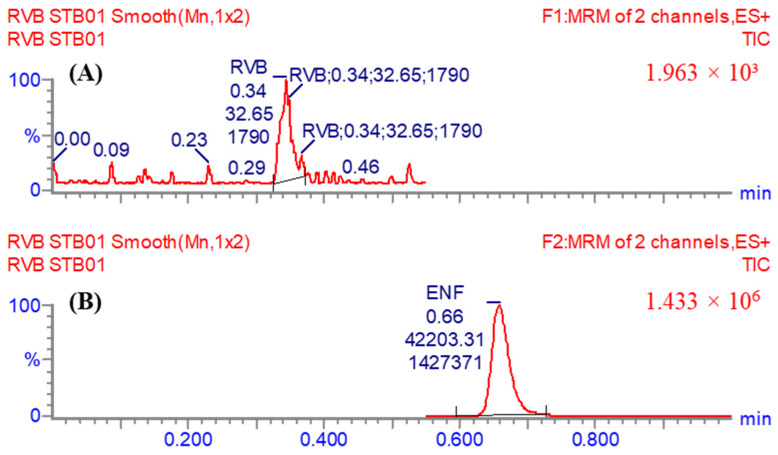
RVB LLOQ chromatographic peak (1 ng/mL) (**A**). The ENF (1000 ng/mL) peak that was used as IS (**B**).

The effectiveness of the established UPLC-MS/MS system was confirmed by its efficacious resolution of chromatographic peaks related to RVB (LLOQ) at 1 ng/mL, as represented in Figure 5. Furthermore, the analysis revealed that the chromatographic peaks associated with the desired compounds, namely RVB and ENF, were not meaningfully influenced by the constituents of the HLM matrix. No detectable residual impact from the previous sample analysis conducted employing RVB was found in the MRM chromatograms obtained from the control samples in the MRM analysis (Figure 4).

#### 3.3.2. Sensitivity and Linearity

Statistical analysis was used to show the linear correlation of the UPLC-MS/MS approach, which spanned a range of 1 to 3000 ng/mL, attained by integrating RVB CSs into the incubation matrix of HLMs; this was then used to determine the results for unknown variables. The variables had strong linear correlation, as indicated by a high coefficient of determination (*R*^2^ = 0.9945) and a linear regression equation of y = 0.6515x − 0.5459. To accommodate the diverse concentration values of the CSs, a weighting factor of (1/x) was applied when constructing the RVB calibration curve. The analysis of Table 3 showed that the RSD of the six repeats, which included both QC and CS samples, was found to be below 3.01%. The LOQ and LOD were estimated to be 0.96 ng/mL and 0.29 ng/mL, respectively (Figure 5).

#### 3.3.3. Precision and Accuracy Validation Features

The evaluation of the precision and accuracy of the established UPLC-MS/MS method was carried out by performing 12 runs, each comprising 4 QCs, in one day. Subsequently, a further six batches, each comprising four QCs, were tested over the following three-day interval. The obtained outcomes were deemed to fall in the designated acceptable range, as stated in the validation measures set forth by the FDA [42]. The implemented UPLC-MS/MS methodology confirmed a variety of precision and accuracy measurements, both in one day and across three consecutive days. The outcomes are displayed in Table 4. The intra-day precision and accuracy spanned from −0.88% to 11.67%, while the inter-day precision accuracy and spanned from −0.23% to 11.33%.

#### 3.3.4. Matrix Effect and Extraction Recovery

The success of the selected protein precipitation extraction approach for RVB and ENF in the UPLC-MS/MS methodology was estimated by performing six repetitions, including four quality controls, using the HLM matrix. Afterwards, the collected data were related to the QCs that were made with the mobile phase. The investigation results demonstrated a substantial level of recovery for RVB extraction (103.53 ± 4.46% with a RSD below 4.31%) and ENF (101.61 ± 3.23% with a RSD lower than 3.18%). An investigation was performed to evaluate the impact of the HLM matrix on ion formation, specifically RVB or ENF. This was done by examining two groups of HLM samples. The findings indicated that the effect was negligible. Sample group 1 was formed by merging the LQC of RVB (3 ng/mL) and ENF (1000 ng/mL), while sample group 2 was formed by switching the HLM incubation matrix with the optimized mobile phase. The matrix of HLMs, composed of RVB and ENF, exhibited an ME of 105.05 ± 5.27% for RVB and 101.68 ± 2.93% for ENF. The normalized ME of the IS was determined to be 1.03, within the allowed threshold set by the controlling standards of the FDA. The experiment’s findings indicated no statistically important relationship between the HLM matrix and the degree of parent ion generation for both ENF and RVB.

#### 3.3.5. Stability

The stability evaluation of RVB in the DMSO and HLM matrices showed that good stability was achieved by maintaining the RVB in DMSO at −80 °C for 28 days. The RSD% for all samples of RVB was consistently less than 2.20% under all working storage settings, as demonstrated in Table 5. There was no considerable drop in the RVB level seen following exposure to long-term storage, auto-sampling, short-term storage, and three freeze–thaw cycles. The results of this experiment provide supportive evidence to verify the good stability RVB.

### 3.4. Assessment of the UPLC-MS/MS Methodology Greenness Employing In Silico AGREE Software

AGREE (v. 0.5 2020), a recently created software application for evaluating environmental sustainability, distinguishes itself by employing the 12 principles of GAC as its input criteria and integrating both qualitative and quantitative factors. The AGREE in silico software is available for free online at the following link: http://www.mostwiedzy.pl/AGREE (accessed on 1 August 2024). The calculator produces a graph that is easily comprehensible to the user and provides a comprehensive score. This tool is often considered more beneficial for determining the environmental sustainability of established analytical methods in comparison to other software packages such as NEMI and AES. The analysis considers the environmental consequences of these methods, including their impact on the analyst and potential sources of risk.

The evaluation of the total environmental sustainability and ecological reliability of the UPLC-MS/MS technique was carried out employing the in silico tool AGREE. The application has been specifically built to include all 12 indicators set by the GAC community [22]. The software utilizes a weighting system that allocates results ranging from 0.0 to 1.0 to different constituents of the GAC methodology. The technique generates analytical scales that are very efficient in assessing the degree of greenness. The data are visually exhibited in a circular pattern which encompasses a wide span of colors, in the range from dark green to red, demonstrating 12 individual qualities.

**Figure 6:**
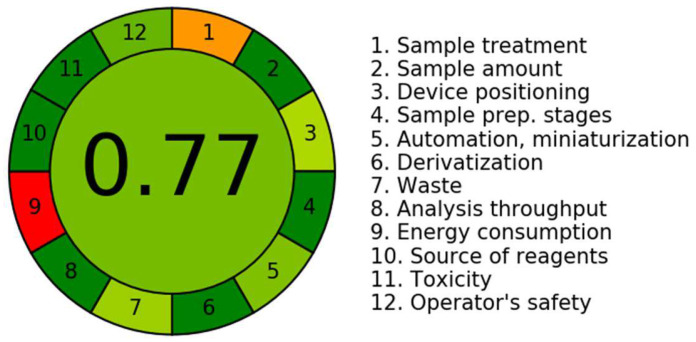
The AGREE programme was employed to demonstrate the greenness scale profile of the established UPLC-MS/MS approach, shown in the form of a circular diagram of twelve separate characteristics.

Figure 6 depicts the degree to which the UPLC-MS/MS procedure demonstrates ecological sustainability. The estimated data for each of the 12 attributes were collected and are presented in Table 6. The existing system was evaluated using various criteria, resulting in a score of 0.77. The generated value serves as a quantitative measure for determining the extent of ecological accountability achieved with the use of the UPLC-MS/MS approach. A score closer to 1.0 suggests a better level of sustainability in the chromatographic procedure. The UPLC-MS/MS approach, a recently created technique, demonstrates a significant degree of eco-friendly accountability, as approved by eco-scale results spanning from 0.75 to 1.00.

### 3.5. In Vitro Metabolic Stability Study of RVB with HLMs

**Figure 7:**
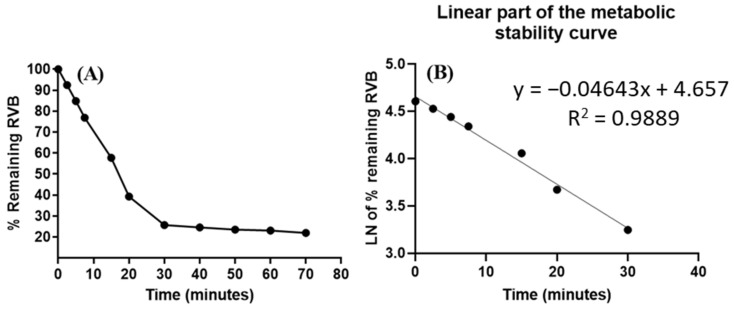
(**A**) RVB metabolic stability curve representing percentage of RVB residual concentration against time intervals; (**B**) linear segment of the metabolic stability curve representing the LN of the percentage of RVB residual level against time intervals, showing the regression equation of the linear part.

The negative control group showed no considerable decrease in the amount of RVB. To estimate the RVB metabolic stability, 1 µM/mL was employed in the in vitro incubation tests with active HLMs matrix. The selection of this specific concentration was made with the objective of keeping it less than the Michaelis–Menten constant, to establish a direct linear link among the rate of RVB metabolism and the length of incubation of in vitro HLMs. In order to reduce non-specific protein binding, 1 mg/mL of HLM protein was used. The fundamental RVB metabolic stability curve was constructed by plotting distinct time intervals for the inhibition of metabolic enzymes, spanning from 0 to 70 min, along the *x*-axis. The *y*-axis in Figure 7A shows the remaining % of RVB.

The linear segment in the previous metabolic stability graph was confined to the designated time range from 0–30 min. The target of this study was to construct a curve explaining the correlation among particular time intervals of incubation (0 to 30 min) and the LN of the ratio of the residual RVB concentration, as depicted in Figure 7B. The study outcomes indicated that the slope (rate of metabolism) of the RVB metabolic stability curve was determined to be 0.04643, as demonstrated by the linear regression equation y = −0.04643x + 4.657, and the linearity was approved as the *R*^2^ value was 0.9889 (Table 7). The in vitro t_1/2_ was computed by applying the formula ln2/slope. The present instance entailed the computation of the in vitro t_1/2_, which was ascertained to be 14.93 min. The measured Cl_int_ of RVB was 54.31 mL/min/kg. McNaney et al. classify RVB as a drug with high clearance, indicating that it can be used without concern for the accumulation of doses in the human body [40]. The use of in silico programs, such as Cloe PK and modeling software, can yield crucial understandings into RVB’s in vivo pharmacokinetics, thereby revealing significant physiological factors [43].

## 4. Conclusions

The current experiment included the launch and assessment of a UPLC-MS/MS approach for quantifying RVB in HLMs. The established methodology was applied for the assessment of RVB metabolic stability. The reported UPLC-MS/MS system revealed significant parameters such as improved sensitivity, selectivity, ecological friendliness, and effective recovery of RVB and ENF from the HLM matrix. The results were obtained by employing ACN as the selected extraction technique to precipitate proteins. The UPLC-MS/MS methodology was developed with a particular emphasis on employing ecologically sustainable techniques, utilizing meticulous protocols. The applied procedures utilized a low flow rate of 0.6 mL/minute and reduced the analysis running time to 1 min. After assessing the environmental sustainability via the AGREE program, it can be inferred that the UPLC-MS/MS method possesses eco-friendly attributes and is perhaps more suitable for routine RVB analysis, without causing any harm to the environment. Based on the data collected, it can be inferred that RVB shares similarities with a highly extracted drug in terms of metabolic stability. Future research should prioritize the comprehensive analysis of RVB’s metabolic profile, specifically identifying the vulnerable metabolic areas, in order to generate more stable derivatives. The existing analytical method should be enhanced by utilizing a more environmentally friendly solvent, such as ethanol. These methods are essential for advancing the development of new pharmaceuticals, particularly in improving metabolic stability.

## Figures and Tables

**Table 1:** ADME characteristics of RVB calculated via the SwissADME online program (freely available).

Physicochemical Features		Water Solubility	
Formula	C_32_H_47_FN_6_O_4_S	Solubility	18.15 × 10^−4^ mg/mL; 1.29 × 10^−6^ mol/L
Heavy atoms	44	Log S (ESOL)	−5.89
Molecular weight	630.82 g/mol	Class	Moderately soluble
Arom. heavy atoms	12	Solubility	2.05× 10^−4^ mg/mL; 3.25× 10^−7^ mol/L
Rotatable bonds	12	Log S (Ali)	−6.49
Fraction Csp3	0.66	Class	Poorly soluble
		Solubility	1.19 × 10^−5^ mg/mL; 1.88 × 10^−8^ mol/L
H-bond donors	1	Log S (SILICOS-IT)	−7.72
H-bond acceptors	9	Class	Poorly soluble
TPSA	116.35 Å^2^	Medicinal Chemistry	
Molar refractivity	177.24	PAINS	0 alert
Lipophilicity		Leadlikeness	No; 3 violations: MW > 350, Rotors > 7, XLOGP3 > 3.5
Log Po/w (XLOGP3)	4.33	Brenk	0 alert
Log Po/w (iLOGP)	4.75	Synthetic accessibility	5.97
Log Po/w (MLOGP)	2.63	Pharmacokinetics	
Log Po/w (WLOGP)	5.42	GI absorption	Low
Log Po/w (SILICOS-IT)	3.32	Permeant to BBB	No
Consensus log Po/w	4.09	P-gp substrate	Yes
Drug likeness		Inhibiton of CYP2D6	No
Lipinski	Yes; 1 violation: MW > 500	Inhibiton of CYP1A2	No
Ghose	No; 3 violations: MW > 480, MR > 130, #atoms > 70	Inhibiton of CYP3A4	Yes
Egan	Yes	Inhibiton of CYP2C9	No
Muegge	No; 1 violation: MW > 600	Inhibiton of CYP2C19	No
Veber	No; 1 violation: Rotors > 10	Skin permeation (Log Kp)	−7.07 cm/s
Bioavailability score	0.55		

**Table 2:** Tuned UPLC-MS/MS Separation Features.

Analytes	Mobile Phase System		Extraction Recovery Yield		Type of Stationary System	
	Methanol	ACN	Solid Phase Extraction	Protein Precipitation Using ACN	C18 Column	C8 Column
RVB	0.79 min	0.36 min	Low (81.83%)	High (103.53 ± 4.46%)	0.84 min	0.36 min
	Tailed and broad	Good peak	Not precise	Precise (RSD < 4.31%)	Tailing of peaks	Good shape
ENF	1.12 min	0.66 min	Good (89.89%)	High (101.61 ± 3.23%	1.86 min	0.66 min
	Superimposed	Optimum peak shape	Not accurate	Accurate (RSD < 3.18%)	Tailing of peaks	Good shape

**Table 3:** Back-calculation data of 6 duplicates of RVB calibration curves (9 CSs).

RVB (ng/mL)	Average	SD	Precision (%RSD)	Accuracy (%E)	% Recovery
1	1.11	0.02	1.87	11.33	111.33
15	16.23	0.39	2.39	8.18	108.18
50	52.96	1.60	3.01	5.91	105.91
150	152.99	4.54	2.96	2.00	102.00
300	292.86	4.34	1.48	−2.38	97.62
400	415.41	8.60	2.07	3.85	103.85
500	489.94	3.70	0.76	−2.01	97.99
1500	1530.01	20.80	1.36	2.00	102.00
3000	3086.33	32.63	1.06	2.88	102.88
% Recovery					103.53 ± 4.46

**Table 4:** Accuracy and precision of the established UPLC-MS/MS system.

RVB (ng/mL)	Intra-Day (12 Groups in the Same Day)				Inter-Day (6 Groups on 3 Consecutive Days)			
QCs	1	3	900	2400	1	3	900	2400
Mean	1.11	3.22	916.03	2394.47	1.12	3.16	918.18	2378.88
SD	0.02	0.04	8.26	8.65	0.03	0.06	9.69	12.26
Precision (%RSD)	1.87	1.35	0.90	0.36	2.88	1.98	1.06	0.52
% Accuracy	11.33	7.33	1.78	−0.23	11.67	5.33	2.02	−0.88
Recovery (%)	111.33	107.33	101.78	99.77	111.67	105.33	102.02	99.12

**Table 5:** RVB stability analysis.

Stability Parameters	3	2400	3	2400	3	2400	3	2400
	Mean		SD		RSD (%)		Accuracy (%)	
Long-Term Stability (28 d at −80 °C)	2.95	2356.31	0.07	10.59	2.20	0.45	−1.56	−1.82
Short-Term Stability (4 hr at room temperature)	3.10	2393.57	0.03	15.31	0.85	0.64	3.33	−0.27
Auto-Sampler Stability (one day at 15 °C)	3.12	2368.44	0.02	12.85	0.67	0.54	3.78	−1.31
Freeze–Thaw Stability (3 cycles at −80 °C)	3.09	2370.79	0.04	8.10	1.31	0.34	3.22	−1.22

**Table 6:** The description data for the present UPLC-MS/MS system according to the GAC guidelines.

Criteria	Score	Weight
1. To circumvent the need of sample handling, it is recommended to utilize straight analytical protocols.	0.3	2
2. The aims of this study are to attain a negligible quantitative representation and a limited sample size.	1.0	2
3. Performance of assessments on-site is highly recommended whenever feasible.	0.66	2
4. Empirical research has demonstrated that the integration of analytical techniques and activities yields favorable outcomes in terms of energy conservation and reagent reduction.	1.0	2
5. Optimal selection of automated and streamlined procedures is recommended.	0.75	2
6. Adopting derivatization techniques should be avoided diligently.	1.0	2
7. Lessening the formation of a substantial amount of analytical waste and implementing efficient disposal techniques are of utmost importance.	0.69	2
8. In the discipline of the field of analytical chemistry, there is a preference for using multi-analyte or multi-factor methods rather than focusing just on a single study.	1.0	2
9. Efforts must be made to decrease energy use.	0.0	2
10. Therefore, it is necessary to arrange the use of reagents derived from maintainable sources.	1.0	2
11. The imperative to eliminate or substitute hazardous chemicals is really substantial.	1.0	2
12. There is a requirement to improve safety ethics for working personnel.	0.8	2

**Table 7:** In vitro metabolic stability data of RVB in HLMs.

Time Intervals (min.)	Average * (ng/mL)	X **	LN X	Linearity Features
0.00	618.73	100.00	4.61	Linear regression line equation: y = −0.04643x + 4.657 *R*^2^ = 0.9889 Slope: −0.04643 t_1/2_: 14.93 min Cl_int_: 54.31 mL/min/kg
2.50	572.18	92.48	4.53	
5.00	524.64	84.79	4.44	
7.50	475.34	76.83	4.34	
15.00	357.19	57.73	4.06	
20.00	243.18	39.30	3.67	
30.00	158.91	25.68	3.25	
40.00	152.13	24.59	3.20	
50.00	145.85	23.57	3.16	
60.00	142.76	23.07	3.14	
70.00	135.79	21.95	3.09	

* Average of 3 repeats. Collision energy. y is the continuous response variable (peak area ratio). X is the nominal value of RVB level. ** X: Mean of the % remaining of RVB level in 3 replicates. *R*^2^: Correlation coefficient.

## Data Availability

The original contributions presented in this study are included in the article. Further inquiries can be directed to the corresponding author.

## References

[1] Mattiuzzi C., Lippi G. (2019). Current Cancer Epidemiology. J. Epidemiol. Glob. Health.

[2] Nehra B., Kumar M., Singh S., Chawla P.A. (2023). Olutasidenib: A ray of hope in the treatment of acute myeloid leukaemia. Health Sci. Rev..

[3] Lin X., Wang J., Huang X., Wang H., Li F., Ye W., Huang S., Pan J., Ling Q., Wei W. (2021). Global, regional, and national burdens of leukemia from 1990 to 2017: A systematic analysis of the global burden of disease 2017 study. Aging.

[4] Bispo J.A.B., Pinheiro P.S., Kobetz E.K. (2020). Epidemiology and etiology of leukemia and lymphoma. Cold Spring Harb. Perspect. Med..

[5] Ning L., Hu C., Lu P., Que Y., Zhu X., Li D. (2020). Trends in disease burden of chronic myeloid leukemia at the global, regional, and national levels: A population-based epidemiologic study. Exp. Hematol. Oncol..

[6] Isidori A., Ferrara F. (2021). The changing landscape for patients with relapsed/refractory acute myeloid leukaemia. Curr. Opin. Oncol..

[7] Bosch F., Dalla-Favera R. (2019). Chronic lymphocytic leukaemia: From genetics to treatment. Nat. Rev. Clin. Oncol..

[8] Greaves M. (2018). A causal mechanism for childhood acute lymphoblastic leukaemia. Nat. Rev. Cancer.

[9] Min H.-Y., Lee H.-Y. (2022). Molecular targeted therapy for anticancer treatment. Exp. Mol. Med..

[10] Sung H., Ferlay J., Siegel R.L., Laversanne M., Soerjomataram I., Jemal A., Bray F. (2021). Global Cancer Statistics 2020: GLOBOCAN Estimates of Incidence and Mortality Worldwide for 36 Cancers in 185 Countries. CA Cancer J. Clin..

[11] Anand U., Dey A., Chandel A.K.S., Sanyal R., Mishra A., Pandey D.K., De Falco V., Upadhyay A., Kandimalla R., Chaudhary A. (2023). Cancer chemotherapy and beyond: Current status, drug candidates, associated risks and progress in targeted therapeutics. Genes Dis..

[12] Ke X., Shen L. (2017). Molecular targeted therapy of cancer: The progress and future prospect. Front. Lab. Med..

[13] Barinaga M. (1997). From Bench Top to Bedside. Science.

[14] Conroy R. (2022). FDA Grants Breakthrough Therapy Designation to Revumenib for Relapsed/Refractory KMT2Ar Acute Leukemia. Cancer Network.

[15] Rim M.H., Karas B.L., Barada F., Dean C., Levitsky A.M. (2024). Recent and anticipated novel drug approvals (Q2 2024 through Q1 2025). Am. J. Health-Syst. Pharm..

[16] Liu M., Shi L., Guo J., Gu Y., Li S., Yi L., Ren D., Li B. (2024). Determination of organic acids for predicting sourness intensity of tea beverage by liquid chromatography–tandem mass spectrometry and chemometrics methods. J. Sep. Sci..

[17] Mahajan B., Miniyar P., Chodankar R., Mahajan A. (2024). Liquid chromatography and liquid chromatography coupled with tandem mass spectrometry studies for the identification and characterization of degradation products of lobeglitazone. Sep. Sci. Plus.

[18] Bosco Ackerman B., Martín S., Espinosa M., Chocrón M., Babay P.A. (2024). Development and validation of a liquid chromatography-mass spectrometry method for quantification of octadecylamine in the secondary circuit of a nuclear power plant in the presence of other amines. Sep. Sci. Plus.

[19] Mudrova B., Hrabakova K., Kozlik P., Hobzova R., Sirc J., Bosakova Z. (2024). A sensitive bioanalytical ultra-high-performance liquid chromatography-tandem mass spectrometry method for the simultaneous quantitation of lactone and carboxylate forms of topotecan in plasma and vitreous. J. Sep. Sci..

[20] Abdelhameed A.S., Kadi A.A., Attwa M.W., AlRabiah H. (2019). Validated LC-MS/MS assay for quantification of the newly approved tyrosine kinase inhibitor, dacomitinib, and application to investigating its metabolic stability. PLoS ONE.

[21] Alrabiah H., Kadi A.A., Attwa M.W., Abdelhameed A.S. (2019). A simple liquid chromatography-tandem mass spectrometry method to accurately determine the novel third-generation EGFR-TKI naquotinib with its applicability to metabolic stability assessment. RSC Adv..

[22] Pena-Pereira F., Wojnowski W., Tobiszewski M. (2020). AGREE—Analytical GREEnness Metric Approach and Software. Anal. Chem..

[23] Duan X., Liu X., Dong Y., Yang J., Zhang J., He S., Yang F., Wang Z., Dong Y. (2020). A Green HPLC Method for Determination of Nine Sulfonamides in Milk and Beef, and Its Greenness Assessment with Analytical Eco-Scale and Greenness Profile. J. AOAC Int..

[24] Goldner D.M., do Nascimento F.H., Masini J.C. (2024). A Green Liquid Chromatography Method for Simultaneous Quantification of Caffeine and its Three Major Metabolites in Urine, Drinks, and Herbal Products. Sep. Sci. Plus.

[25] Marothu Vamsi K., Kantamaneni P., Gorrepati M., Katherine D. (2021). In vitro Metabolic Stability of Drugs and Applications of LC-MS in Metabolite Profiling. Drug Metabolism.

[26] Houston J.B. (1994). Utility of in vitro drug metabolism data in predicting in vivo metabolic clearance. Biochem. Pharmacol..

[27] Obach R.S., Baxter J.G., Liston T.E., Silber B.M., Jones B.C., MacIntyre F., Rance D.J., Wastall P. (1997). The prediction of human pharmacokinetic parameters from preclinical and in vitro metabolism data. J. Pharmacol. Exp. Ther..

[28] Kontostathi G., Makridakis M., Bitsika V., Tsolakos N., Vlahou A., Zoidakis J. (2019). Development and validation of multiple reaction monitoring (MRM) assays for clinical applications. Proteom. Biomark. Discov. Methods Protoc..

[29] Busby W.F., Ackermann J.M., Crespi C.L. (1999). Effect of methanol, ethanol, dimethyl sulfoxide, and acetonitrile on in vitro activities of cDNA-expressed human cytochromes P-450. Drug Metab. Dispos..

[30] Störmer E., Roots I., Brockmöller J. (2000). Benzydamine N-oxidation as an index reaction reflecting FMO activity in human liver microsomes and impact of FMO3 polymorphisms on enzyme activity. Br. J. Clin. Pharmacol..

[31] Fouin-Fortunet H., Tinel M., Descatoire V., Letteron P., Larrey D., Geneve J., Pessayre D. (1986). Inactivation of cytochrome P-450 by the drug methoxsalen. J. Pharmacol. Exp. Ther..

[32] Magdy G., Al-enna A.A., Belal F., El-Domany R.A., Abdel-Megied A.M. (2024). Quality-by-design optimized RP-HPLC approach for the therapeutic drug monitoring of glibenclamide and fluoxetine in human plasma. Microchem. J..

[33] Smith G. (2012). European Medicines Agency guideline on bioanalytical method validation: What more is there to say?. Bioanalysis.

[34] Zhang D., Liu B., Xiao T., Wang Y., Zhao Z., Xie J.A., Li W., Li R., Cui J. (2024). Development and validation of a simultaneous quantitative analytical method for two Alternaria toxins and their metabolites in plasma and urine using ultra-high-performance liquid chromatography-tandem mass spectrometry. J. Sep. Sci..

[35] Attwa M.W., Abdelhameed A.S., Kadi A.A. (2024). An Ultra-Fast Green UHPLC-MS/MS Method for Assessing the In Vitro Metabolic Stability of Dovitinib: In Silico Study for Absorption, Distribution, Metabolism, Excretion, Metabolic Lability, and DEREK Alerts. Medicina.

[36] Alsubi T.A., Attwa M.W., Bakheit A.H., Darwish H.W., Abuelizz H.A., Kadi A.A. (2020). In silico and in vitro metabolism of ribociclib: A mass spectrometric approach to bioactivation pathway elucidation and metabolite profiling. RSC Adv..

[37] Gosetti F., Mazzucco E., Zampieri D., Gennaro M.C. (2010). Signal suppression/enhancement in high-performance liquid chromatography tandem mass spectrometry. J. Chromatogr. A.

[38] Ghosh C., Shinde C.P., Chakraborty B.S. (2012). Influence of ionization source design on matrix effects during LC-ESI-MS/MS analysis. J. Chromatogr. B Anal. Technol. Biomed. Life Sci..

[39] AlRabiah H., Kadi A.A., Attwa M.W., Mostafa G.A.E. (2020). Development and validation of an HPLC-MS/MS method for the determination of filgotinib, a selective Janus kinase 1 inhibitor: Application to a metabolic stability study. J. Chromatogr. B Anal. Technol. Biomed. Life Sci..

[40] McNaney C.A., Drexler D.M., Hnatyshyn S.Y., Zvyaga T.A., Knipe J.O., Belcastro J.V., Sanders M. (2008). An automated liquid chromatography-mass spectrometry process to determine metabolic stability half-life and intrinsic clearance of drug candidates by substrate depletion. Assay Drug Dev. Technol..

[41] Słoczyńska K., Gunia-Krzyżak A., Koczurkiewicz P., Wójcik-Pszczoła K., Żelaszczyk D., Popiół J., Pękala E. (2019). Metabolic stability and its role in the discovery of new chemical entities. Acta Pharm..

[42] Meesters R., Voswinkel S. (2018). Bioanalytical method development and validation: From the USFDA 2001 to the USFDA 2018 guidance for industry. J. Appl. Bioanal..

[43] Leahy D.E. (2006). Integrating invitro ADMET data through generic physiologically based pharmacokinetic models. Expert Opin. Drug Metab. Toxicol..

